# Isolation and Characterization of a Baculovirus Associated with the Insect Parasitoid Wasp, *Cotesia* *marginiventris*, or Its Host, *Trichoplusia ni*

**DOI:** 10.1673/031.008.4201

**Published:** 2008-05-22

**Authors:** James J. Grasela, Arthur H. McIntosh, Kent S. Shelby, Steve Long

**Affiliations:** Biological Control of Insects Research Laboratory, USDA-ARS 1503 S. Providence Road, Research Park, Columbia, Missouri, 65203-3535 USA

**Keywords:** TnMNPV/CmBCL9, *polh*, egt, *p10*, AfMNPV, *Anagrapha falcifera*, split-graphs network

## Abstract

A multiple nucleopolyhedrovirus (MNPV) was isolated from *Trichoplusia ni* (Hübner) (Lepidoptera: Noctuidae) larvae that had been stung by the parasitoid *Cotesia marginiventris* (Cresson) (Hymenoptera: Braconidae). The wild type virus was plaque purified by infecting a *Heliothis subflexa* (BCIRL- HsAM1) cell line and isolating several clones. The mean estimated genomic size of this virus based on *Pst*I, *BstE*II, *Sty*I, *Hind*III restriction profiles was estimated to be 106 ± 2.5 kbp (mean±SE). A clone designated as TnMNPV/CmBCL9 was used in bioassays against several lepidopteran pests and in comparative studies with the baculoviruses AcMNPV, AgMNPV, AfMNPV, PxMNPV and HzSNPV of *Autographa califomica, Anticarsia gemmatalis, Anagrapha falcifera, Plutella xylostella*, and *Helicoverpa zea*, respectively. Infectivity studies showed that TnMNPV/CmBCL9 was highly infectious for *Heliothis subflexa* and *T. ni*, with an LC_50_ value 0.07 occlusion bodies/mm^2^ in both species and also infectious for *H. zea* and *Heliothis virescens* with LC_50_ values of 0.22 and 0.27 occlusion bodies/mm^2^, respectively. Restriction endonuclease analysis of the isolate and selected baculoviruses revealed profiles that were very similar to AfMNPV but different from the restriction endonuclease profiles of the other baculoviruses. Hybridization studies suggest that the TnMNPV/CmBCL9 was closely related to AfMNPV and AcMNPV-HPP. Further support for this comes from a phylogenetic analysis employing a split-graphs network, comparing the *polh, egt, and p10* genes from TnMNPV/CmBCL9 with those from other baculoviruses and suggests that this virus is closely related to the AcMNPV variants, AfMNPV and RoMNPV of *Rachiplusia ou*.

## Introduction

Baculoviruses are double stranded DNA viruses belonging to the family Baculoviridae that infect members of the phylum Arthropoda, mainly insects from the order Lepidoptera, but also other insect orders including Hymenoptera, Diptera, Coleoptera, Neuroptera, Thysanura and Trichoptera. They have also been reported to occur in the order Decapoda (shrimp) (Tanada and Kaya 1993; Possee 1993; Murphy et al. 1995). Baculoviruses have been successfully used worldwide to control Lepidopteran and Hymenopteran insect pests of agriculture and forestry importance (Granados and Federici 1986; Miller 1997; Moscardi 1999) and thus help reduce the need for chemical insecticides.

The Baculoviridae family is comprised of two genera, the *Nucleopolyhedrovirus* and the *Granulovirus* (Murphy et al. 1995). Members of these genera, such as the multiple and single nucleopolyhedroviruses (MNPV, SNPV), and granuloviruses, have a unique biphasic replicative cycle in which budded virus is produced early in the infection, and later, when viral particles are produced, they become occluded into proteinacious occlusion bodies formerly referred to as polyhedral inclusion bodies. The budded virus is responsible for the systemic spread of the virus within the host and is the entity used for infecting cell culture. The occlusion bodies are the main means by which the virus is disseminated in the environment between susceptible larvae. This is achieved through cell lysis of infected larvae resulting in contamination of the leaf surfaces and subsequent consumption of leaf tissue by healthy larvae.

There are many reports on the association of insect viruses with parasitoid wasps belonging to the families Braconidae and Ichneumonidae (Stoltz and Vinson 1977, 1979; Stoltz and Faulkner 1978; Vinson and Iwantsch 1980 A; Vinson and Iwantsch 1980 B; Fleming et al. 1983; Styer et al. 1987; Strand and Pech 1995; Doucet and Cusson 1996; Ferrarese et al. 2005). Such association may be as a contaminant on the parasitoid, or the virus may be internalized in the host tissues as is the case with the polydnaviruses that are the most studied (Kroemer and Webb 2004; Webb and Strand 2005).

The objectives of the present report were to establish the identity of the baculovirus isolated from parasitized *T. ni* larvae, to determine the relationship of this isolate to other well known baculoviruses, and to attempt to determine the possible origin of the newly isolated baculovirus.

## Materials and Methods

### History of the parasitoid

The braconid parasitoid, *Cotesia marginiventris* was originally obtained from the USDA, ARS, Stoneville, MS facility where it was reared on *Spodoptera exigua*. At the time, no indication of a possible baculovirus infection in the colony was reported. After receivership the parasitoid was then initially reared on *Spodoptera frugiperda* larvae obtained periodically from the USDA, ARS, Starkville, MS with no observable baculovirus symptoms reported either from that facility nor later at our laboratory. The parasitoid was then reared on *Tnchoplusia ni* larvae available inhouse from our insectary and there was no report of an observed baculovirus infection in the *T. ni* colony subsequent to exposure to the parasitoid.

### Recovery and propagation of a baculovirus from Cotesia *marginiventris*

In the course of immunological studies employing *C. marginiventris*, it was found that several *T. ni* larvae that were stung by this parasitoid displayed typical baculovirus symptoms resulting in lysis of the larvae. Examination by light microscopy of the liquid contents from *T. ni* cadavers revealed the presence of occlusion bodies. *T. ni* larvae displaying typical baculovirus infection were consistently observed on other occasions following parasitization. Occlusion bodies from collected dead larvae were fed to 3rd instar *T. ni* by topical application to a wheatsoy diet (Bio-Serv, www.bio-serv.com) surface in order to amplify occlusion bodies as well as serve as a source of infectious hemolymph for inoculation of cell cultures.

### Determination of possible latent viral infection in *T*. *ni larvae*

To investigate the possibility that individuals in the *T. ni* colony might harbor TnMNPV/CmBCL9 as a latent virus, 35 early 3^rd^ instar *T. ni* larvae from the laboratory colony were stressed by incubating them at 37°C for six days to monitor for any pathogenic signs of an infection that would indicate a possible latent virus.

### Viral source originating from the adult parasitoid interior

To investigate a possible viral source originating internally from the parasitoid, ten *C. marginiventris* from an exteriorly washed group of 40 insects resulting in *T. ni* infection were macerated in 2 ml Hanks' Balanced Salt Solution (HBSS) (Sigma, Co., www.sigmaaldrich.com), spun at 10,000 rpm in a tabletop centrifuge for 5 min to remove insect debris and then passed through a 0.22 µn filter. 30 µl undiluted samples of this filtrate were added to each of 15 wells of a 50-well tray each containing artificial diet and a 2^nd^ instar *T. ni* larva. An equivalent number of larvae were used as controls. They were then incubated at 28°C to monitor for larval pathogenicity. Another 30 µl sample of undiluted filtrate was also used to inoculate three T-25 cm^2^ flasks (5 ml) containing 1 × 10^5^ cells/ml to determine possible budded virus presence in the parasitoid. Another flask containing the same TN-CLl cell concentration was mock infected to act as a control.

### Viral source originating from surface contact with a contaminated adult parasitoid

The question of whether or not the virus could have been transmitted through surface contact with an exteriorly contaminated parasitoid was also investigated. Forty adult parasitoids were collected and initially stored at -80^°^C. One ml of HBSS was added to the sample and then stored at 4°C for several days. The intention was to have the solution gently remove any potential parasitoidsurface virus so that it could be used as inoculum for both *in vitro* and *in vivo* assays. For the *in vitro* assay, 1 ml inoculum sterilized through a 0.22 µm filter was added to a T-25 cm^2^ flask containing about 1 × 10^5^ TN-CL1 cells/ ml. The inoculum was removed after 2 h and replaced with 5 ml ExCell-401 (10% FBS) medium and incubated at 28°C. Another flask containing the same TN-CL1 cell concentration was mock infected to act as a control. To test whether the virus was present as occlusion bodies attached to the parasitoid body surface, 30 µl of surfacewashed parasitoid solution was added to each of 15 wells of a 50-well tray each containing artificial diet and a 2nd instar *T. ni* larva. An equivalent number of larvae were used as controls. Trays were then incubated at 28°C to monitor for larval pathogenesis.

### Plaque purification of wild type virus

Infectious hemolymph was collected from five 3^rd^ instar *T. ni* larvae fed approximately 10^5^ occlusion bodies and hemolymph collected on ice 48 h after exposure by snipping several prolegs. The infectious hemolymph was diluted at a ratio of 1:2 with ExCell 401 (SAFC Biosciences, www.sigmaaldrich.com/SAFC/Biosciences.htm1) and passed through a 0.45 µm millipore filter. A T-25 cm^2^ flask of *Heliothis subflexa* cells (BCIRL-HS-AM1, McIntosh 1991) at 4 × 10^5^ cells/ml in 5 ml of ExCell 401 containing 10% inactivated fetal bovine serum and antibiotics (McIntosh et al. 2005) were inoculated with 0.5ml of the filtered infectious hemolymph and incubated at 28°C for 5 days. Supernatant fluid was recovered by centrifugation at 1500 × g for 10 min and the cell pellet containing occlusion bodies were re-suspended in 5 ml of purified water and stored at -20°C. The supernatant from the infected BCIRL-HS-AM1 cell line was used to plaque purify the virus as previously described (McIntosh et al. 1997) and clones isolated. Selected clones and wild type virus were produced in 3 T-225 cm^2^ flasks in BCIRL-HS-AM1 cells and the budded virus collected for DNA extraction (McIntosh et al. 2005).

### Restriction enzyme analysis

Restriction endonuclease analyses were performed on the wild type virus as well as on selected clones that gave identical profiles. One of the clones, (TnMNPV/ CmBCL9), was selected for a comparative study of its restriction endonuclease profile with those of several other baculoviruses and was used in all the remaining studies. The nucleopolyhedroviruses employed were: *Autographa* *californica* (AcMNPV) (McIntosh and Ignofo 1989), *Anagrapha falcifera* (AfMNPV), (McIntosh 1991), *Anticarsia gemmatalis* (AgMNPV), (Grasela and McIntosh 1998), *Plutella xylostella* (PxMNPV), (Kariuki et al. 2000) and the single nucleopolyhedrovirus from *Helicoverpa zea* (HzSNPV), (McIntosh et al. 2001). These baculoviruses were produced in cell culture as described and DNA extracted from budded virus as previously reported (McIntosh and Grasela 2006). The restriction enzymes used included *Hin*dIII, *Sty*I, *Vsp*I, *Bst*EII, *Xho*I and *Pst*I and digestion of the DNA was carried out according to the manufacturers instructions.

### Hybridization studies

Hybridization studies were conducted with the 5 named baculoviruses as well as TnMNPV/CmBCL9 to determine the relationship if any, of the latter with the known baculoviruses. A previously described protocol (Kariuki and McIntosh 1999) using random primed DNA labeling of a *Vsp*I probe with digoxigenin-dUTP was followed for this comparative study.

### Electron microscopy

Samples of TnMNPV/CmBCL9 occlusion bodies were prepared for transmission electron microscopy as previously described (Kariuki and McIntosh 1999) to determine whether the virus was a SNPV or MNPV, and were processed by the Electron Microscopy Core facility at the University of Missouri-Columbia.

### *In vivo* infectivity studies of TnMNPV/ CmBCL9 occlusion bodies

Occulsion bodies produced in BCIRL-HS-AM1 were used in infectivity studies against 24h old larvae from *T. ni, S. frugiperda, S. exigua, Helicoverpa zea, Heliothis virescens*, and *H. subflexa*. Both *T. ni* and *H. subflexa* larvae were obtained from the insectary at the Biological Control of Insects laboratory and the remaining larvae were obtained commercially (Bio-Serv, www.bio-serv.com). For each virus tested, the sample size consisted of three groups of 25 larvae per dosage replicated twice. Larvae were incubated at 28°C for 7 days, and mortalities were recorded daily for all insects and the LC_50_ values calculated employing PoloPlus v.l (LeOra Software, leorasoftware.com).

### Determining the DNA sequences of the polyhedrin, egt, and *p10* genes

Polyhedrin protein sequences from *Autographa califomica* MNPV (AcMNPV) (GenBank No. NC_001623), *Anticarsia gemmatalis* MNPV (AgMNPV) (GenBank No. NC_008520), *Bombyx mori* MNPV (BmMNPV) (GenBank No. L33180), *Spodoptera frugiperda* MNPV (SfMNPV) (Genbank No. AY250076), and *Orgyia pseudotsugata* MNPV (OpMNPV) (GenBank No. U75930) were used with the web-based software Block Marker and the algorithm MOTIF (Smith et al. 1990) (http://blocks.fhcrc.org/blocks/make_blocks.html) to find conserved blocks in the related five unaligned protein sequences. The blocks were then used with the algorithm CODEHOP (COnsensus-DEgenerate Hybrid Oligonucleotide Primers) (Rose et al. 1998) (http://blocks.fhcrc.org/codehop.html) to generate the following pair of degenerate primers used to PCR amplify a predicted 651 bp fragment of the TnMNPV/CmBCL9 polyhedrin gene:

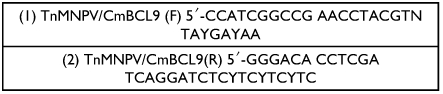

A similar approach was also taken to determine theTnMNNPV/CmB *egt* sequence employing degenerate primers designed from the following protein sequences: AcMNPV, AgMNPV_,_ *Helicoverpa armigera* MNPV (HaMNPV) (Genbank No. NC_003094), OpMNPV, *Rachiplusia ou* (RoMNPV) 4 (GenBank No. NC_004323), and SfMNPV. A predicted 1629 bp egt fragment was generated using the following primer pair:

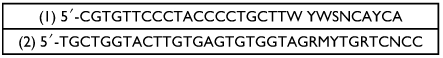

Likewise, a partial *p10* sequence was determined by employing the following degenerate primers based on the p10 amino acid sequences of AcMNPV, AgMNPV, BmMNPV, OpMNPV, and RoMNPV to generate a 204 bp p10 fragment:

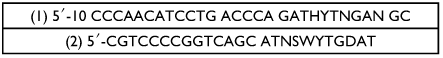

TnMNPV/CmBCL9 DNA (100 –200 ng/µl) was amplified using puReTaq Ready-To-Go PCR beads (Amersham Biosciences, www.apbiotech.com) under the following conditions: 95°C, 3 min (1X); 94°C, 45 s, 60°C, 1 min, 72°C, 2 min (40X); 72°C, 5 min; held 15°C. The reaction products were run on a 2% Metaphor gel containing 1 µg/µl ethidium bromide and visualized with a VersaDoc imaging system (Bio-Rad Laboratories, Inc., www.bio-rad.com). The expected amplicon product was gel extracted using QIAEX II gel extraction kit (Qiagen, Inc., www.qiagen.com). Purified amplicon products were then cloned into the pCR4-TOPO plasmid according to the protocol provided by the manufacturer (Invitrogen, Corp., www.invitrogen.com). To obtain a more reliable nucleotide sequence read of the putative TnMNPV/ CmBCL9 *polyhedrin* gene, two clones, labeled TnMNPV/ CmBCL1 and TnMNPV/CmBCL2, containing the amplicon from two separate PCR reactions were sequenced from the 5′- and the 3′-end of the amplicon insert using M13 Forward (-20) and M13 Reverse primers by the DNA Core facility at the University of Missouri. The four sequence reads were subsequently used to generate a consensus sequence of the TnMNPV/CmBCL9 polyhedrin gene employing the BioEdit Sequence Editor (Hall 1999).

### Nucleotide sequence accession numbers

The *polh, egt, and p10* sequences described in this study have been deposited in GenBank; the accession numbers are EF418027 EF418026, and EF418025, respectively.

### Analysis of sequence data

Multiple-sequence alignmenst of the nucleotide sequences were performed using T-Coffee (Notredame et al. 2000), which generates a library of the best global and local alignments based on the Sim algorithm from the Lalign package (Huang and Miller 1991). The CORE index was employed to evaluate the consistency between a multiple alignment and every pair of aligned residues contained in the library. To obtain a more accurate picture of the relationship between the newly isolated TnMNPV/CmBCL9 and other viruses in the Baculoviridae family, a network-based tool was employed for deciphering the evolutionary relationships in molecular sequence data. The method builds a network primarily constructed from distances determined from splitdecomposition theory and can be implemented using the SplitsTree4 (v.4.6) program that generates a Split-graphs network (Huson 1998; Huson and Bryant 2006). A network-based approach has several advantages, one of which avoids the implicit assumption of a tree-like evolutionary process. This allows one the flexibility to determine if the data follow a tree-like evolutionary path or to identify some other underlying pattern the typical tree representation might not discern. The significance of the topology of the split-graphs was verified by bootstrap resampling (1000 replicates). The program allows one to choose among various distance matrixes to construct a graphical representation of the phylogenetic relationship.

## Results and Discussion

### Electron microscopy

**Figure 1.**
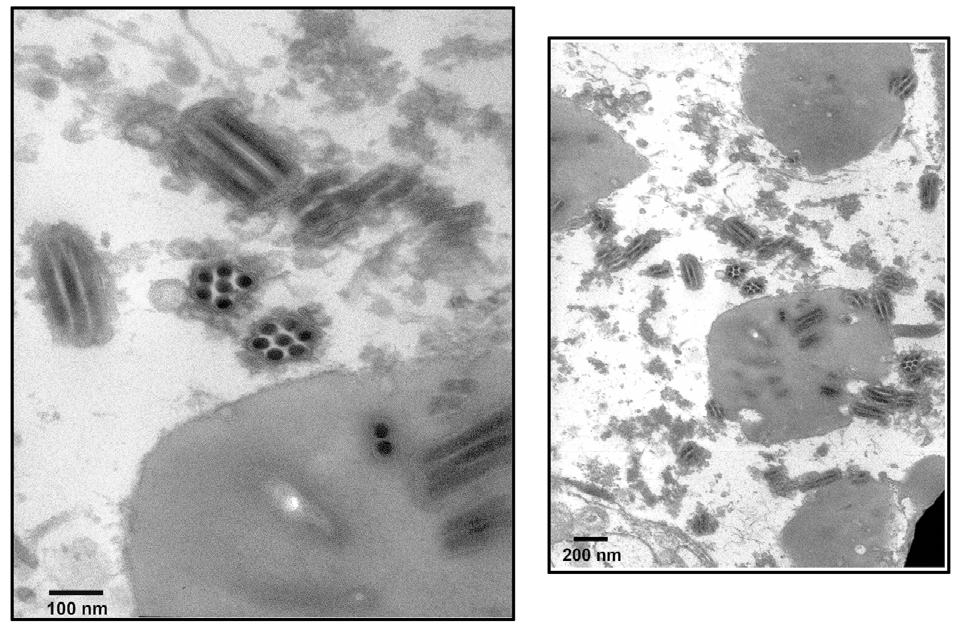
Transmission electron micrograph of the TnMNPV/CmBCL9 in BCIRL-HS-AM1 cells.

Transmission electron microscopy of TnMNPV/ CmBCL9 occlusion bodies are depicted in Figure 1. It shows the virus to be of the MNPV type because they contain many buddle-like structures embedded within a polyhedrin protein matrix each containing a multiple number of smaller virus particles.

### Latent viral presence in the *T. ni* larvae

Of the 35 3^rd^ instar larvae reared at 37°C, only five larvae died after six days of exposure. None of these larvae as well as the remaining insect showed any pathology typical of a baculovirus infection. This suggests that the source of the virus might come more from contamination rather than a possible latent virus.

### Viral source originating from the adult parasitoid interior

All 15 2^nd^ instar larvae treated with a solution from macerated parasitoid showed no sign of pathogenicity after 7 days incubation. It is recognized that this test would only detect budded virus because the sample was passed through a 0.22 µm filter. It is also known that early instar larvae are not the most sensitive system for assaying for budded virus. There was no observed mortality in an equivalent number of control larvae. TN-CL1 cells inoculated with the filtered macerated parasitoid solution showed no signs of occlusion bodies formation after 7 days, other than general deterioration of cells probably from some component in the HBSS wash. Control cells were normal and almost confluent after 5 days.

### Viral source originating from contact with parasitoid

TN-CL1 cells inoculated with the parasitoid-surface wash showed no sign of occlusion bodies formation after 7 days other than general deterioration of cells probably from some component in the HBSS wash. Control cells were normal and almost confluent after 5 days. When the supernatant collected from these cells was used to inoculate a fresh batch of TN-CL1 cells, no signs of viral infection were observed. Additionally, all 2^nd^ instar larvae fed on diet treated with the parasitoid-surface wash also showed no pathogenesis nor did the untreated controls after 7 days. It is highly unlikely that budded virus would remain stable on the surface of an insect long enough to be somehow transmitted. Occlusion bodies attached to the parasitoid body that contaminate the diet surface would be the more plausible source of infection

### Restriction endonuclease analysis and molecular weight of TnMNPV/CmBCL9

**Figure 2.**
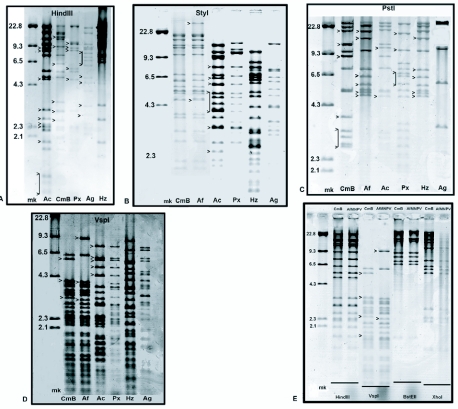
Comparison of five restriction profile patterns between TnMNPV/CmBCL9 and other baculoviruses. (A) *Hin*dIII restriction profile: (mk) DNA marker in kb; (Ac) AcMNPV-HPP; (CmB) TnMNPV/CmBCL9; (Px) PxMNPVCL3; (Ag) AgMNPV-CL4-3A1; and (Hz) HzSNPV. (B) *Sfy*I restriction profile: (mk) DNA marker in kb; (CmB) TnMNPV/CmBCL9; (Af) AfMNPV; (Ac) AcMNPV-HPP; (Px) PxMNPVCL3; (Hz) HzSNPV; and (Ag) AgMNPV-CL4-3A1. (C) *Pst*I restriction profile: (mk) DNA marker in kb; (CmB )TnMNPV/ CmBCL9; (Af) AfMNPV; (Ac) AcMNPV-HPP; (Px) PxMNPVCL3; (Hz) HzSNPV; and (Ag) AgMNPV-CL4-3A1; (D) *Vsp*I restriction profile: (mk) DNA marker in kb; (CmB) TnMNPV/CmBCL9; (Af) AfMNPV; (Ac) AcMNPV-HPP; (Px) PxMNPVCL3; (Hz) HzSNPV; and (Ag) AgMNPV-CL4-3A1: (E) Comparison of four different restriction profile patterns between TnMNPV/CmBCL9 and AfMNPV.

Two of the plaque purified clones (TnMNPV/CmBCL9 and TnMNPV/CmBCL14) and wt TnMNPV/CmB gave identical *Hin*dIII restriction patterns (data not shown). TnMNPV/CmBCL9 was employed in all other studies. A comparison of the *Hin*dIII restriction pattern between TnMNPV/CmBCL9 and four other baculoviruses revealed a number of differences both in the number as well as molecular size of the DNA fragments (Figure 2A). Six major bands ranging from 10.0 kb to 2.3 kb were found unique to TnMNPV/CmBCL9, whereas several *Hin*dIII restriction fragments present in AcMNPV-HPP (from 2.1 kb to 9.3 kb), PxMNPVCL3 (4.5 kb, 5.5 kb, 9.1 kb, 9.2 kb, 9.4 kb, 9.5 kb), AgMNPCL4-3A1 (from 6.0 kb to 9.3 kb) were absent in TnMNPV/CmBCL9. Most of the smaller *Hin*dIII bands (< 4.3 kb) of HzSNPV were absent in TnMNPV/ CmBCL9 as well as in the other viruses examined. A comparison of the *Sty*I fragmentation profile showed a significant number of differences between TnMNPV/ CmBCL9 and AcMNPV-HPP, PxMNPVCL3, AgMNPCL4-3A1, and HzSNPV (Figure 2B). In contrast, the overall *Sty*I restriction profile between TnMNPV/CmBCL9 and AfMNPV were indistinguishable except for the presence of two unique restriction fragments in AfMNPV. No further restriction pattern differences were detected on examination of these two viruses with the enzymes *Hin*dIII, *Bst*EII, and *Xho*I in terms of identical number and molecular size distribution of fragments (Figure 2C). However, the *Vsp*I restriction pattern revealed major differences between TnMNPV/ CmBCL9 and AfMNPV especially in the higher molecular size region (> 6.5 kb) as well as three unique bands between the 4.3 – 6.5 kb region of AfMNPV (Figure 2D). A number of major band differences in the *Pst*I restriction profiles were also evident among TnMNPV/CmBCL9, AcMNPV-HPP, PxMNPVCL3, AgMNPV-CL1-3A1, and HzSNPV (Figure 2E). In particular, there were six unique *Pst*I fragments in AfMNPV that were absent in TnMNPV/CmBCL9, whereas eight unique bands were detected in TnMNPV/CmBCL9 that were absent in AfMNPV. In contrast, two unique *Pst*I fragments that were detected in AcMNPV were absent in TnMNPV/CmBCL9. The molecular weight of TnMNPV/CmBCL9 was estimated to be 106 ± 2.5 kbp (mean ± SE) based on *Pst*I, *Bst*EII, *Sty*I, *Hin*dIII restriction profiles. This compares with an estimated mean genomic size for AfMNPV of 118.1 Kbp S.E. ± 6.9 in the host *T. ni* (Vail et al. 1993).

**Figure 3.**
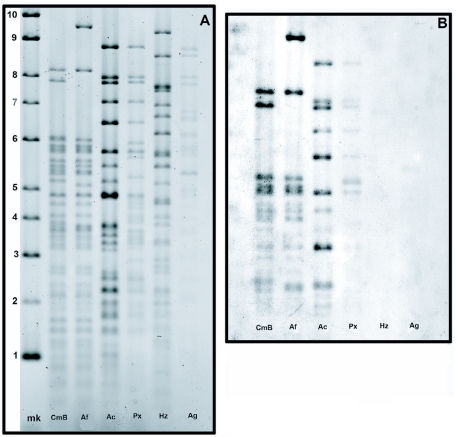
(A) Comparison of *Vsp*I restriction patterns between TnMNPV/CmBCL9 and five other baculoviruses. (mk) DNA marker in kb; (CmB) TnMNPV/CmBCL9; (Af) AfMNPV; (Ac) AcMNPV-HPP; (Px) PxMNPV-C13; (Hz) HzSNPV; and (Ag) AgMNPV-CL4-3A1. (B) Southern hybridization using a dig-labeled *Vsp*I probe from TnMNPV/CmBCL9. (CmB) TnMNPV/CmBCL9; (Af) AfMNPV; (Ac) AcMNPV-HPP; (Px) PxMNPVCL3; (Hz) HzSNPV; (Ag) and AgMNPV-CL4-3A1.

### Hybridization studies

The *Vsp*I restriction profile showed some clearly distinct band differences specifically within the 6–10 kb range between TnMNPV/CmBCL9 and the other viruses. Hybridization analysis employing a *Vsp*I probe constructed from genomic TnMNPV/CmBCL9 revealed that the restriction pattern of the TnMNPV/CmB isolate appears to be more genetically similar to AcMNPV-HPP and AfMNPV than to PxMNPV, AgMNPV-CL1-3A1, or HzSNPV (Figure 3A, B). Federici and Hice (1997), also showed AfMNPV to be a genomic variant of AcMNPV based on Southern hybridization, the organization of the polyhedrin gene region, and nucleotide and deduced amino acid sequences of eight other viral genes in this region.

### *In vivo* infectivity studies of TnMNPV/ CmBCL9 occlusion bodies

Analysis of the hypothesis of equality and parallelism showed that changes in TnMNPV/CmBCL9 infectivity per unit change in dosage rate were significantly different among the six species tested (P< 0.001) (Table 1). However, viral activity at a specific response level may still be similar between some comparisons. For example, the TnMNPV/CmB CL9 virus was equally effective against *H. zea* (LC_50_ = 0.22 occlusion bodies/mm^2^) and *H. virescens* (LC_50_ = 0.26 occlusion bodies/mm^2^). TnMNPV/CmBCL9 showed a 4.2 -12.2X lower infectivity against *S. exigua* larvae in comparison to reported LC_50_s of *Plutella xylostella* MNPV (CL3) (0.70 occlusion bodies/ mm^2^), AcMNPV (2.01 occlusion bodies/mm^2^), and AfMNPVCL1 (1.67 occlusion bodies/mm^2^) (Kariuki and McIntosh 1999). The LC_50_ of TnMNPV/CmBCL9 in *S.Jrugiperda* was 13.1 occlusion bodies/mm^2^. Although they used 2nd-instar larvae incubated at 26°C and recorded an accumulative mortality after a 10-day period, Berretta et al. (1997) reported that *S. frugiperda* larvae infected with SfMNPV-AR and SfMNPV-ME isolates from Argentina had LC_50_s (13.9 and 14.0 occlusion bodies/mm^2^, respectively) similar to the TnMNPV/CmB CL9 LC_50_ reported here. Of the six species tested, the TnMNPV/CmBCL9 virus was most virulent against *H. subflexa* (LC_50_ = 0.07 occlusion bodies/mm^2^) and *T. ni* (LC_50_ = 0.07 occlusion bodies/mm^2^). Given that TnMNPV/CmB CL9 appears to be a variant of both AcMNPV and AfMNPV some further comparison can be made between TnMNPV/CmBCL9 and these two viruses. Although they used neonates instead of 24 h-old larvae, Hostetter and Puttler (1991) found AcMNPV and AfMNPV LC_50_ values to be somewhat higher for *H*. *virescens* (0.45 occlusion bodies/mm^2^ and 0.35 occlusion bodies/mm^2^, respectively) and *T. ni* (0.39 occlusion bodies/mm^2^ and 0.15 occlusion bodies/mm^2^, respectively) relative to TnMNPV/CmBCL9. Finally, a major difference can be seen in TnMNPV/CmB CL9 where this virus is more infectious in *H. zea* (LC_50_ = 0.22 occlusion bodies/mm^2^) than AcMNPV (10.3 occlusion bodies/mm^2^) as reported by Hostetter and Putler (1991).

**Table 1.**
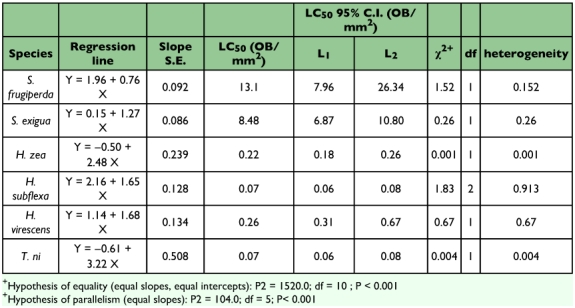
Probit analysis results of larval mortalities from six lepidopteran species infected with TnMNPV/CmBCL9.

### Analysis of the *polh*, *egt*, and p10 sequences

The distribution of BLAST hits showed that the TnMNPV/CmB CL9 polyhedrin nucleotide sequence had the best E values when aligned with RoMNPV (E = 0.0) and AfMNPV (E = 0.0). The TnMNPV/CmB CL9 *polyhedrin* sequence has a GC content of 46.7%, while the AfMNPV *polh* gene (Genbank no. AFU64896) was reported to have a GC content of 46.1%. The multiplesequence alignment of the TnMNPV/CmB CL9 polyhedrin nucleotide and predicted protein sequences with seven viruses selected from the Genbank (BmMNPV [U75359], AfMNPV [AAB53357], OpMNPV [M14885], AcMNPV [KO1149], RoMNPV [DQ345451], SfMNPV [JO4333], AgMNPV [NC_008520]) is depicted in Figure 4A. If in the alignment only the partially determined TnMNPV/CmBCL9 sequence based on 651 nucleotides is considered, then the nucleotide composition between the polyhedrin sequence of TnMNPV/CmB CL9 and the other viruses (BmMNPV, AfMNPV, OpMNPV, AcMNPV, RoMNPV, SfMNPV, AgMNPV) consist of sequences having a 21.7 identity. If one makes a similar comparison using aligned amino acids sequences, then the residue composition between the TnMNPV/CmB CL9 *polh* gene and the other viruses consist of sequences having a 74.2% identity, and a 2.2%, 12.4% semi-conserved and conserved substitution level, respectively. Comparatively, the BLAST distribution is somewhat incomplete as there are no records of either the *egt* or the *p10* genes from AfMNPV in the GenBank database, and, as such, these comparisons are tentative. The TnMNPV/CmBCL9 *egt* has a GC content of 45.1% and the *p10* a GC content of 36.7%. For the partial aligned *egt and p10* nucleotide sequences, 33.0% and 4.8 identities, respectively, were found when TnMNPV/CmBCL9 was compared with six different viruses: BmMNPV, OpMNPV, AcMNPV, RoMNPV, SfMNPV, AgMNPV (Figure 5A, Figure 6A). For the partial *egt* and *p10* aligned protein sequences the semi-conserved substitution levels were 10.9% and 1.2%, respectively and conserved substitutions were 20.6% and 13.1%, respectively. Initial split-graph analysis of the *polh* nucleotide and translated protein sequences clearly placed TnMNPV/CmB CL9 into a group consisting of RoMNPV/AfMNPV/BmMNPV and this relationship can be explained for the most part as a split-graph network rather than a tree-like network (Figure 7). The central portions of the *egi* (Figure 8) *and p10* (Figure 9) graphs also show box-like structures that indicate incompatible data suggesting a network-like rather than a simple evolutionary tree structure. The PHI test of recombination showed significant evidence for recombination in the *polh* aligned sequences (p = 1.894 × 10^-5^). In contrast, no evidence was found to indicate a recombination signal in either the *egt* (p = 0.1052) nor *p10* (p = 0.1549) sequences. The split graphs of the *egt* and *p10* DNA sequences are illustrated in Figure 8 and Figure 9, respectively. Parallel evolution, model heterogeneity, and sampling error along with recombination can result in misleading interpretation of phylogenetic histories. Baculoviruses are known for exhibiting recombination and their evolutionary histories may not be best represented by a bifurcating or multifurcating trees. We chose the use of a split-graph network as its premise is that it can represent a relationship among lineages without assuming a tree-like evolutionary process. Also a major difference between a network and a tree is that cycles are permitted in which paths start and end at the same node. In general, similar network patterns were generated between each of the DNA and protein aligned sequences of the three genes (Figures 7, 8, 9). Based on the *polh* and *egt* sequence alignments TnMNPV/CmB CL9 was grouped with RoMNPV, BmMNPV, AfMNPV, and AcMNPV.

**Figure 4A.**
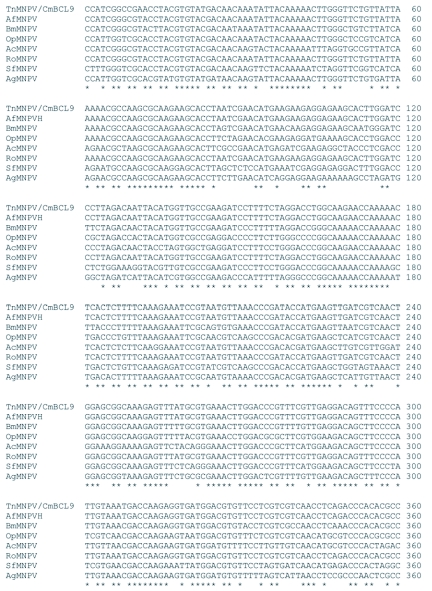
Multiple-sequence alignment of the TnMNPV/CmBCL9 *polh* nucleotide sequence with seven baculoviruses employing the T-Coffee package, (*) indicate identical residues.

**continued:**
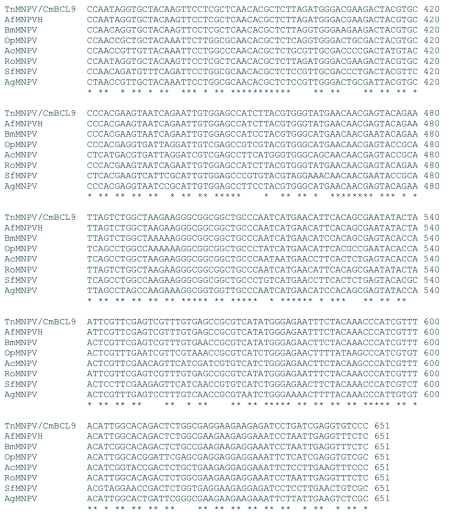

**Figure 4B.**
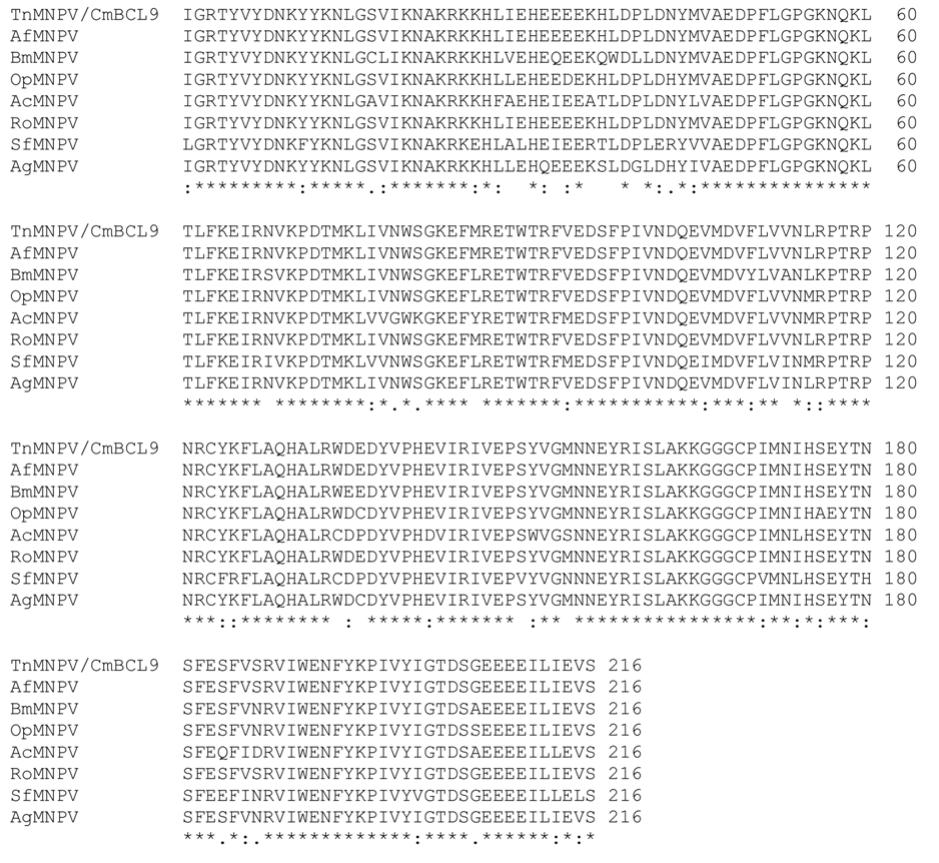
The putative amino acid sequence of the TnMNPV/CmBCL9 *polh* gene: (*) indicate identical residues; (:) indicate semiconserved residues; (.) designate conserved residues.

Specifically, the AfMNPV node in Figure 7A might be interpreted as the ancestor of TnMNPV/CmBCL9. In the absence of a published AfMNPV *egt* gene sequence, analysis without AfMNPV still grouped TnMNPV/ CmBCL9 with RoMNPV, AcMNPV, and BmMNPV. On the other hand, based on *the p10* gene network, the relation of TnMNPV/CmBCL9 with the other viruses was counter to what the *polh* and *egt* gene network showed by placing TnMNPV/CmBCL9 as a distantly unique virus (Figure 9). When all three gene sequences were concatenated using all the viruses except AfMNPV, TnMNPV/CmBCL9 was still grouped with RoMNPV and BmMNPV.

**Figure 5A.**
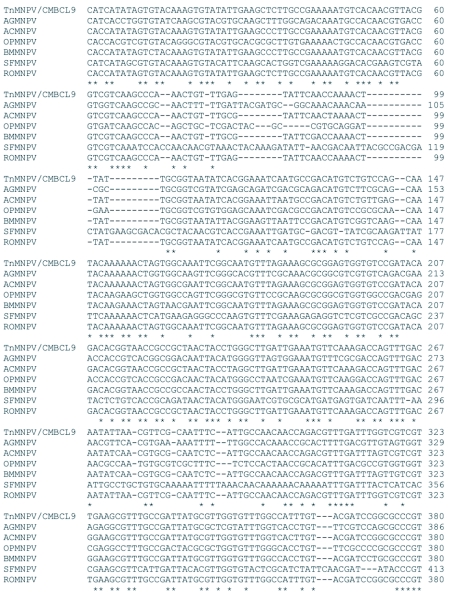
Multiple-sequence alignment of the TnMNPV/CmBCL9 *egt* nucleotide sequence with six baculoviruses employing the TCoffee package, (*) indicate identical residues.

**continued:**
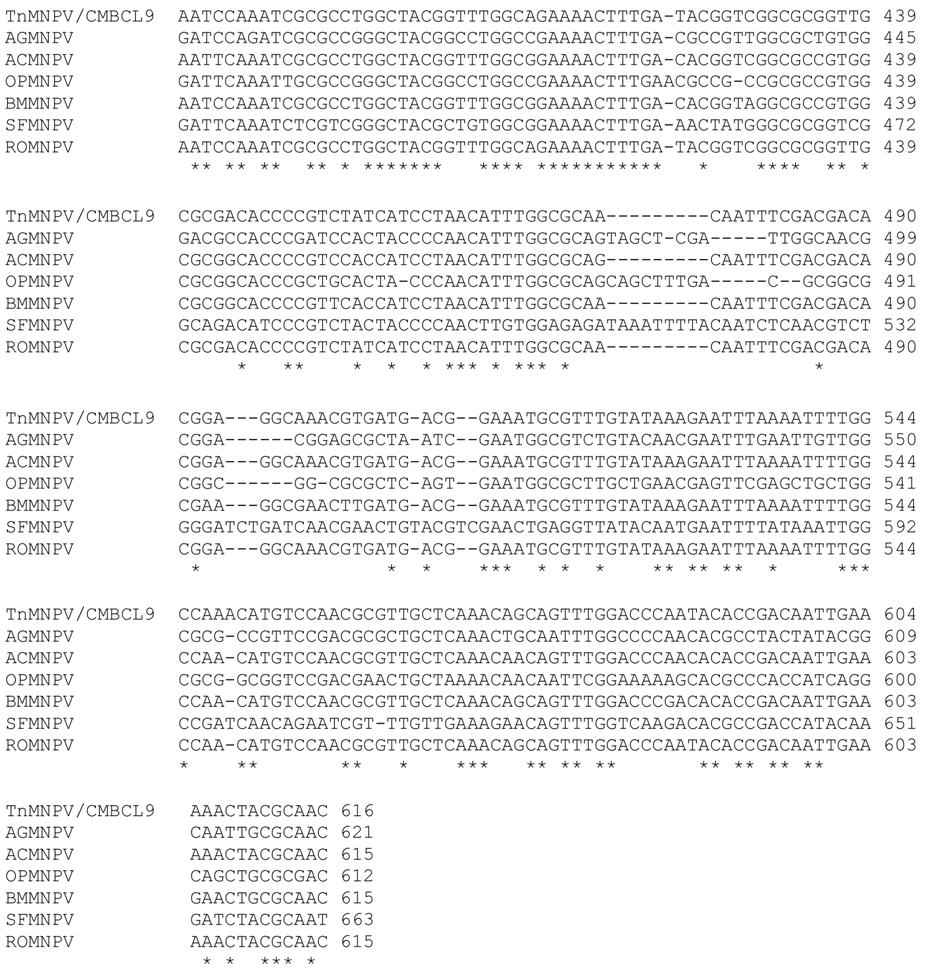

In this study, information is presented on a new multiple nucleopolyhedrovirus variant, TnMNPV/CmB CL9 found in *T. ni* larvae following parasitization with the parasitoid *C. marginiventris*. Larval mortality studies showed that the virus is highly infectious for 24 h-old *T*. *ni* and *H. subflexa* larvae and infectious for both 24 h-old *H. zea* and *H. virescens* larvae. Restriction DNA and hybridization profiles indicated that TnMNPV/CmB CL9 is genetically similar to AfMNPV, but appears to be a new isolate of the multiple nucleopolyhedrovirus type. The partially determined *polh, egt, and p10* nucleotide sequences in a split-graph analysis further demonstrated a close relationship to AfMNPV, BMNPV, and RoMNPV, previously determined genetic variants of AcMNPV. Various possible sources of the virus that were examined in this study included (1) surface contamination of parasitoid, (2) virus sequestered by the parasitoid, and (3) a latent virus present in the *T. ni* colony. The latter possibility seems unlikely since no infection of larvae was observed in the stock colony of *T. ni* and attempts to activate latency were unsuccessful. The actual origin of the virus at this time remains unknown.

**Figure 5B.**
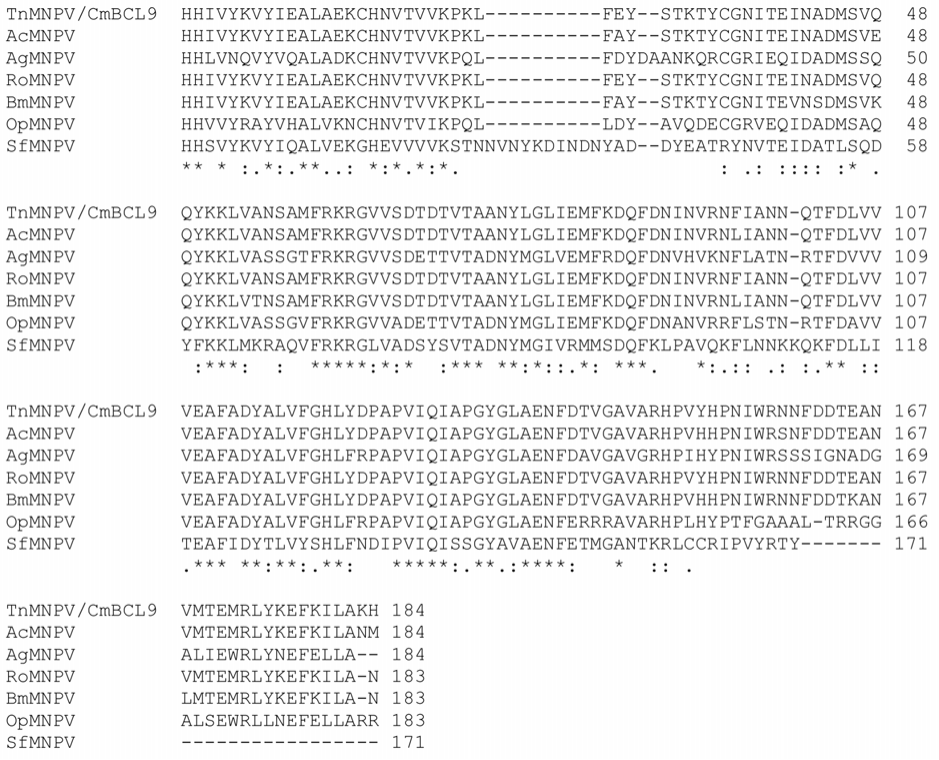
The putative amino acid sequence of the TnMNPV/CmBCL9 *polh* gene: (*) indicate identical residues; (:) indicate semiconserved residues; (.) designate conserved residues.

**Figure 6A.**
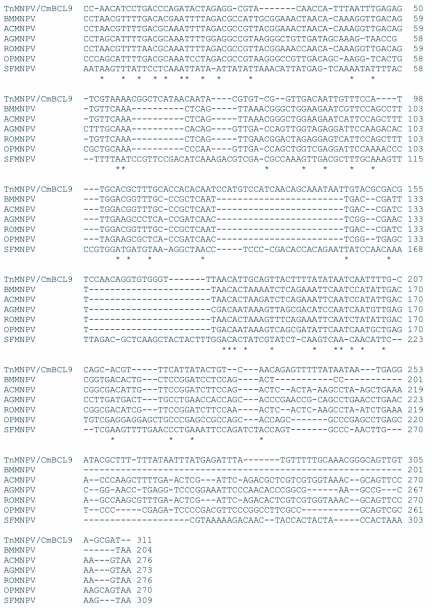
Multiple-sequence alignment of the TnMNPV/CmBCL9 *p10* nucleotide sequence with five baculoviruses employing the T-Coffee package. (*) indicate identical residues.

**Figure 6B.**
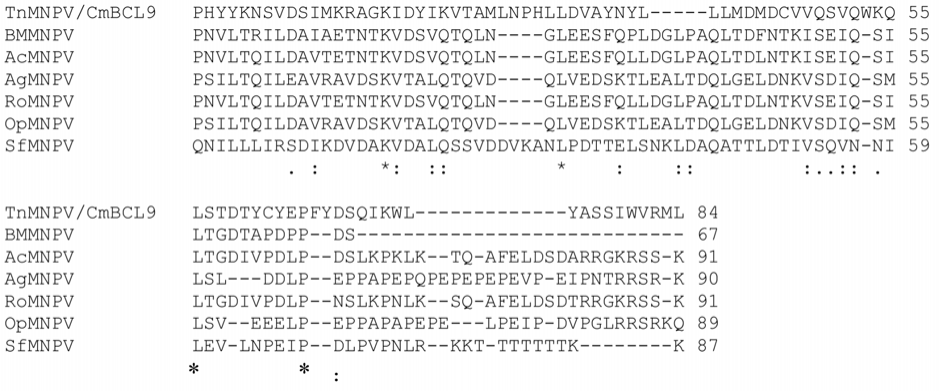
The putative amino acid sequence of the TnMNPV/CmBCL9 *polh* gene: (*) indicate identical residues; (:) indicate semiconserved residues; (.) designate conserved residues.

**Figure 7.**
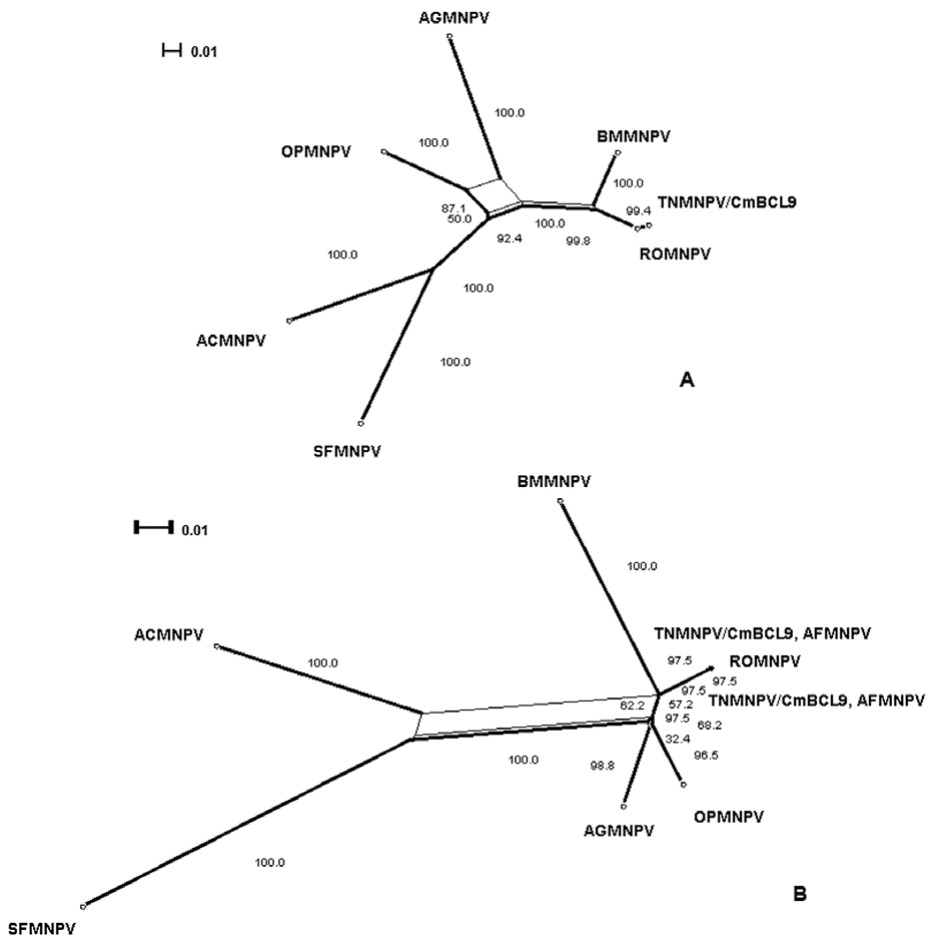
The split-graph of the *polh*, egt, and *p10* genes shows that the phylogenetic relationship among the viruses is more appropriately depicted by a non-tree like data set (box-like structures) rather than the typical bifurcating tree. The Hamming distances (d_h_), which are computed by giving equal weight to the number of nucleotide differences as well as insertions-deletions between each sequence pair between taxa are accurately represented by the split-graph based on a fit index of 91.7. The number besides each branch is the percent of computed graphs in which a split corresponding to the branch occurred by bootstrap re-sampling.

**Figure 8.**
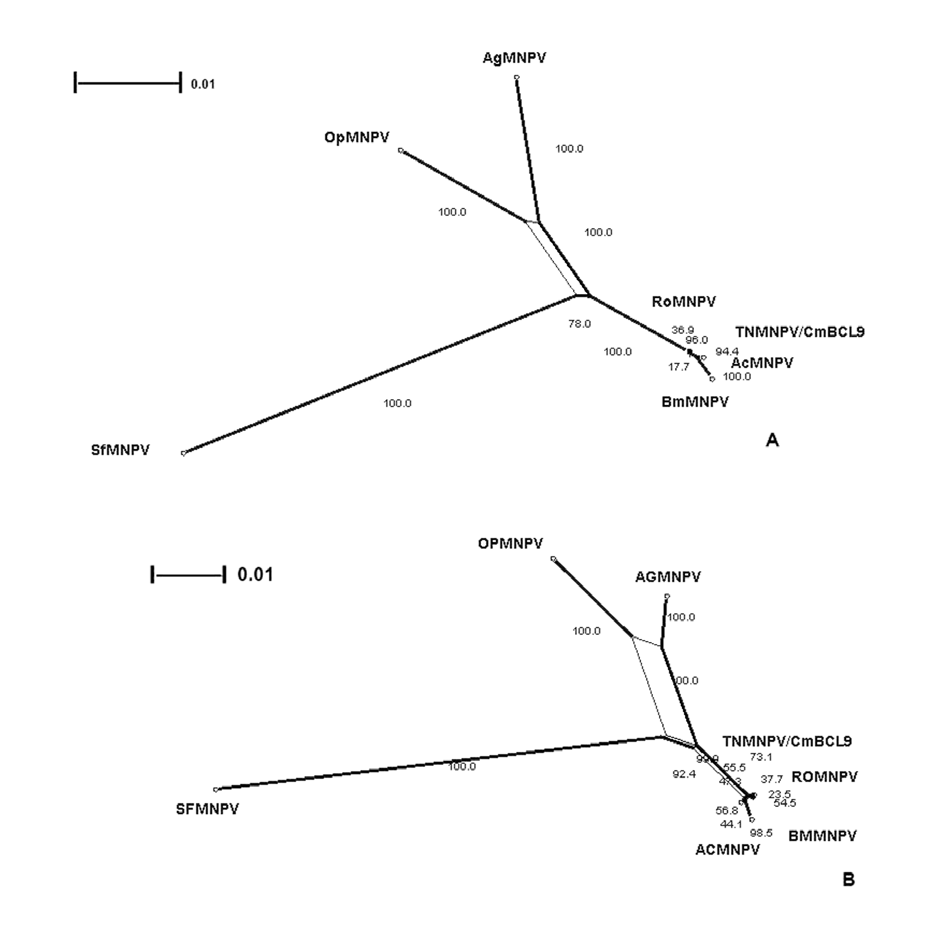
The split-graph of aligned *egt* DNA sequences from seven baculovirus 33 including TNMNPV/CmBCL9. This network is based on a logDet character transformation matrix and split-decomposition distance transformation. The fit index 35 indicates that 95% of the original distance is represented by the split-graph. (B) The split-graph of the aligned *egt* protein translated DNA sequences from seven 37 baculovirus including TNMNPV/CmBCL9. The split index is 98.1%. The splits were calculated employing the ProteinMLdist character transformation and split- 39 decomposition distance transformation.

**Figure 9.**
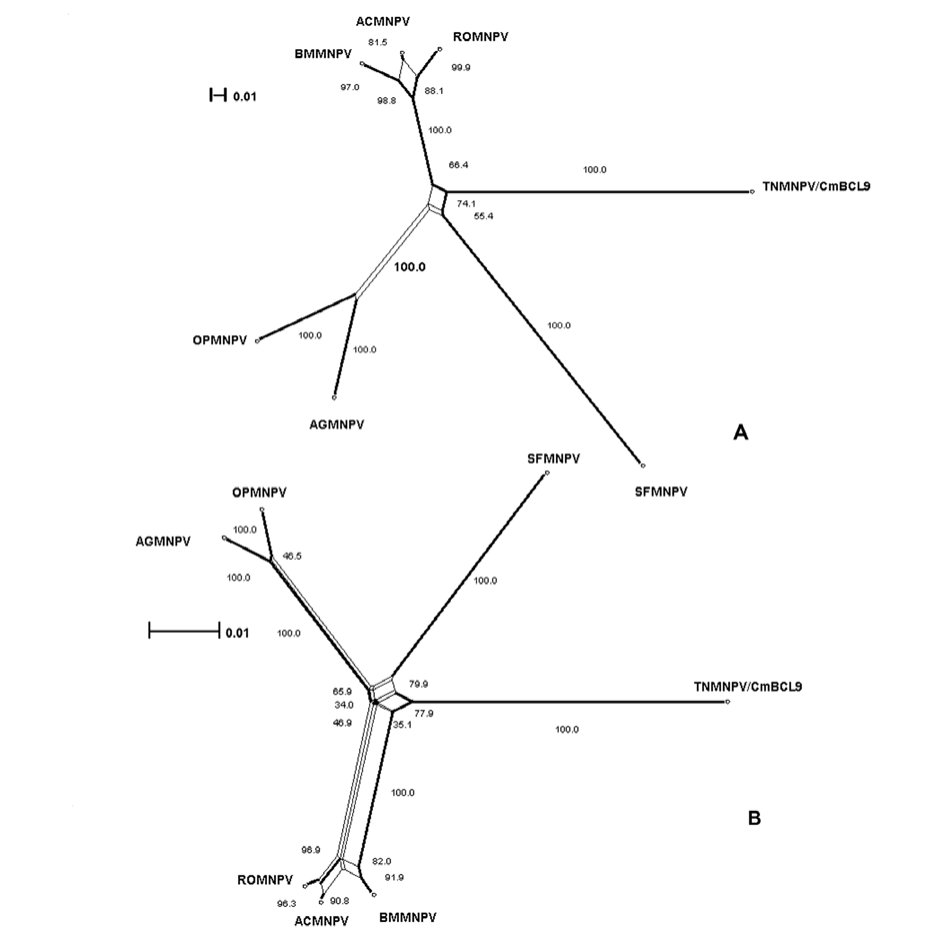
The split-graph of aligned *p10* DNA sequences from seven baculovirus including TNMNPV/CmBCL9. This network is based on a uncorrected_P character 43 transformation matrix and split-decomposition distance transformation. The fit index is 13 indicates that 90.4% of the original distance is represented by the split-graph. (B) I The split-graph of the aligned *p10* protein translated DNA sequences from seven baculovirus including TNMNPV/CmBCL9. The split index is 98.7%. The splits were 3 calculated employing the uncorrected_P character transformation and splitdecomposition distance transformation.

## Abbreviations

HzSNPV -: Helicoverpa zea single nucleopolyhedroviruses
TnMNPV -: Trichoplusia ni multiple nucleopolyhedroviruses
wt -: wildtype
